# Pto Interaction Proteins: Critical Regulators in Plant Development and Stress Response

**DOI:** 10.3389/fpls.2022.774229

**Published:** 2022-03-10

**Authors:** Meihong Sun, Liuliu Qiu, Yanshuang Liu, Heng Zhang, Yongxue Zhang, Yi Qin, Yingjie Mao, Min Zhou, Xiaosha Du, Zhi Qin, Shaojun Dai

**Affiliations:** Development Center of Plant Germplasm Resources, College of Life Sciences, Shanghai Normal University, Shanghai, China

**Keywords:** Pto interaction (Pti), protein kinase, transcription factor, disease resistance, stress response

## Abstract

Pto interaction (Pti) proteins are a group of proteins that can be phosphorylated by serine/threonine protein kinase Pto, which have diverse functions in plant development and stress response. In this study, we analyzed the phylogenetic relationship, gene structure, and conserved motifs of Pti1s and predicted the potential *cis*-elements in the promoters of *Pti1* genes using bioinformatics methods. Importantly, we systematically summarized the diverse functions of Pti1s in tomato, rice, *Arabidopsis*, potato, apple, and cucumber. The potential *cis*-elements in promoters of Pti1s decide their functional diversity in response to various biotic and abiotic stresses. The protein kinase Pti1 was phosphorylated by Pto and then modulated the downstream signaling pathways for PTI and ETI in the disease insistence process. In addition, some transcription factors have been defined as Ptis (e.g., Pti4, Pti5, and Pti6) originally, which actually were ethylene-response factors (ERFs). Pti4, Pti5, and Pti6 were modulated by salicylic acid (SA), jasmonate (JA), and ethylene signaling pathways and regulated diverse defense-related gene expression to cope with Pst infection and insect wounding.

## Introduction

Pto interaction protein (Pti) refers to the proteins that can directly interact with the resistance (R) protein Pto (Zhou et al., 1995). Pti mainly referred to the homologous proteins of tomato SlPti1. Tomato SlPti1 is the first protein that has been screened to interact with Pto (Zhou et al., 1995), which belongs to the receptor-like cytoplasmic kinase (RLCK) family involved in immunity defense to *Pseudomonas syringae* pv. Tomato (Pst) (Zhou et al., 1995). In *Arabidopsis*, there are 11 homologous genes of tomato *SlPti1* (Anthony et al., 2006), all of which encode serine/threonine kinases and participate in plant growth and development, lipid metabolism, oxidative stress response, and other biological processes (Zhou et al., 1995; Shiu and Bleecker, 2001a,b, 2003; Anthony et al., 2006; Forzani et al., 2011; Liao et al., 2016). Additionally, the members of ethylene-response factor (ERF) family were previously defined as Pti4, Pti5, and Pti6 (Zhou et al., 1995; Gu et al., 2002). ERF is a group of transcription factors, such as SlPti4, SlPti5, and SlPti6 in tomato, which participate in defense responses by regulating the expression of pathogenesis-related (*PR*) genes (Zhou et al., 1997; Gu et al., 2002).

It has been reported that Pti proteins were critical for plant disease resistance, ROS homeostasis, and diverse abiotic stress responses (Gu et al., 2002; Anthony et al., 2006; Takahashi et al., 2007; Forzani et al., 2011). In this study, we systematically analyzed the structural characteristics and evolutionary relationships of these Pti family members. Moreover, we summarized the roles of Pti proteins in pathogen-associated molecular patterns (PAMP)-triggered immunity and effector-triggered immunity (ETI) of disease resistance, oxidative stress, and other abiotic stresses, as well as growth and development. This provides valuable information for further understanding the Pti functions.

### Conservation of Pti1 Kinases Revealed From Phylogenetics, Gene Structure, and Motifs Analyses

**FIGURE 1:**
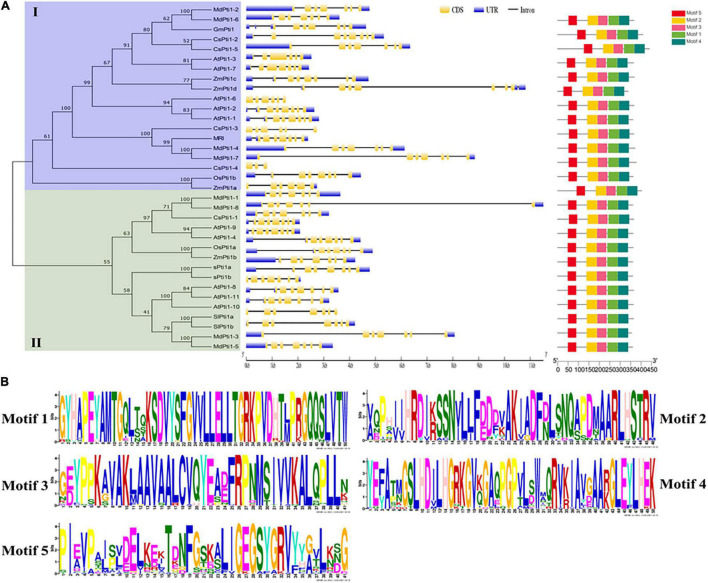
Phylogenetic relationships, gene structures, and conserved motifs analysis of Pti1s. **(A)** The analysis of phylogenetic relationships, gene structures, and conserved motifs in the Pti genes from *Solanum lycopersicum* (Sl), *Arabidopsis thaliana* (At), *Glycine max* (Gm), *Oryza sativa* (Os), *Malus domestica* (Md), *Cucumis sativus* (Cs), and *Zea mays* (Zm). The phylogenetic tree was constructed based on the full-length protein sequences of the 34 Pti1 proteins using MEGA 7.0 software. In the analysis of the gene structure, the number indicates the phases of corresponding introns. The UTR, exon, domain, and motif are displayed in different colors, and the intron is displayed in a straight line. **(B)** The logos indicate the conserved motifs in the 34 Pti1 proteins.

To identify the putative Pti1s in *Arabidopsis thaliana*, soybean (*Glycine max*), rice (*Oryza sativa*), maize (*Zea mays*), apple (*Malus domestica*), and cucumber (*Cucumis sativus*), the protein sequence of SlPti1a in tomato (*Solanum lycopersicum*) was used as a query and submitted to the BlastP program for searching NCBI database.^1^ A total of 11 *Arabidopsis* AtPti1s, four maize ZmPti1s, two rice OsPti1s, three soybean GmPti1s, eight apple MdPti1s, and five cucumber CsPti1s were obtained with default parameters. Taken together with the two tomato SlPti1s, a total of 34 Pti1s were used to perform protein sequence alignment and phylogenetic analyses (Supplementary Table 1). The phylogenetic comparison showed that these Pti1s were divided into two groups based on their protein sequence homology, namely, group I and group II (Figure 1A). Among them, six members of *Arabidopsis* AtPti1s, namely, AtPti1-1, AtPti1-2, AtPti1-3, AtPti1-6, AtPti1-7, and MARIS (MRI); four members of apple MdPti1s, namely, MdPti1-2, MdPti1-4, MdPti1-6, and MdPti1-7; and rice OsPti1b were classified into group I. All cucumber CsPti1s belong to group I except CsPti1-1. GmPti1 belongs to group I, while the other two Pti1s of soybean, namely, sPti1a and sPti1b, belong to group II. Three Pti1s, namely, ZmPti1b, ZmPti1c, and ZmPti1d, belong to group I, while only one Pti1, namely, ZmPti1b, belong to group II. Group II also contained AtPti1-4, AtPti1-8, AtPti1-9, AtPti1-10, and AtPti1-11 of *Arabidopsis*; MdPti1-1, MdPti1-3, MdPti1-5, and MdPti1-8 of apple; and OsPti1a of rice. Tomato SlPti1a and SlPti1b were distributed in group II.

The motif analysis of 34 Pti1 proteins indicated that five distinct motifs were involved in all the kinases (Figure 1A). The five motifs were all located at PKc-like superfamily domain, a conserved kinase domain in Pti1 proteins, which catalyze the transfer of the γ-phosphoryl group from ATP to hydroxyl groups in specific substrates. Therefore, the five motifs are proposed to be essential for Pti1 protein kinase function (Wu et al., 2018). The conserved amino acid sites in the five motifs were shown in Figure 1B, which implied their potential importance for the kinase activity. Among them, only the threonine (Thr) site in the motif2 has been reported to be a critical conserved phosphorylation site for the interaction between Pti1 and Pto (Sessa et al., 2000; Matsui et al., 2010b). The mutation of Thr^233^ in tomato SlPti1 abolished the Pti1-Pto interaction in the yeast two-hybrid system (Sessa et al., 2000). Similarly, the Thr^233^ at OsPti1 was phosphorylated by OsOXI1 for positively mediating the rice resistance to blast fungus, while the mutation of Thr^233^ at OsPti1 led to the disease susceptibility (Matsui et al., 2010b).

### Potential *Cis*-Elements in Promoters of *Pti1* Genes Imply Their Functional Diversities

**FIGURE 2:**
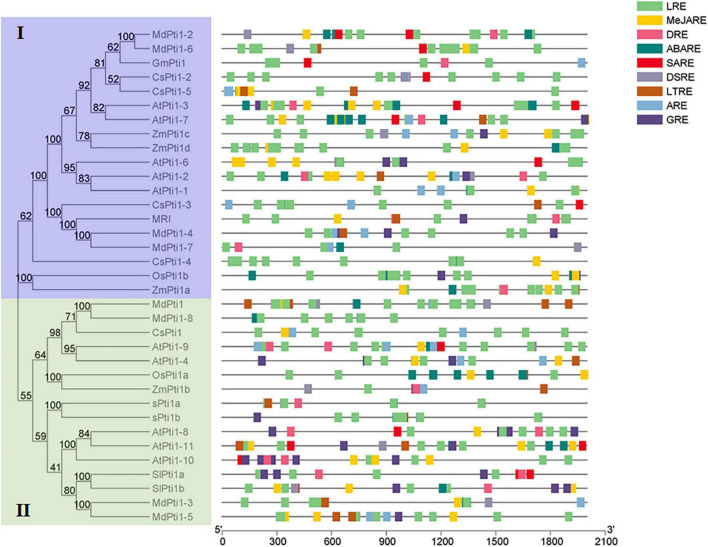
*Cis*-elements in the promoter regions of *Pti1* genes. The stress-responsive *cis*-elements are distributed on the gray line. The MeJARE, DSRE, GRE, ABARE, LRE, LTRE, DRE, SARE, and ARE sequences are, respectively, represented by rectangles of different colors. MeJARE, MeJA-responsive element; DSRE, defense and stress-responsive element; GRE, gibberellin-responsive element; ABARE, abscisic acid-responsive element; LRE, light-responsive element; LTRE, low-temperature-responsive element; DRE, drought-responsive element; SARE, salicylic acid-responsive element; ARE, auxin-responsive element.

It has been reported that Pti1 proteins were involved in the regulation of plant development and stress tolerance. To evaluate the possible function of *Pti1* genes, the 2.5-kb promoter regions of all the 34 *Pti1* genes from *Arabidopsis*, rice, maize, soybean, apple, cucumber, and tomato were submitted to predict the potential *cis*-elements using the Plant CARE database.^2^ In the results, the *cis*-elements are implied to be involved in the responses to low temperature, light, drought, defense, and stress; and diverse phytohormones [e.g., abscisic acid (ABA), gibberellin, salicylic acid (SA), auxin, and methyl jasmonate (MeJA)] were overrepresented in the promoters (Figure 2). Several of these speculations of *cis*-elements were proved by gene functional analyses. *Arabidopsis AtPti1*-*4* was induced by H_2_O_2_ treatment for 1 h, implying it was involved in oxidative stress (Forzani et al., 2011). Besides, it has been reported that several *Pti1s* from different plant species were in response to various stresses, such as high salts-, drought-, and low temperature-induced maize Zm*Pti1* (Zou et al., 2006); wounding-increased soybean *GmPti1* (Tian et al., 2004); and salt-elevated cucumber *CsPti1-L* (Oh et al., 2014). Additionally, SA-induced *ZmPti1* and *GmPti1*, and MeJA-, SA-, and ABA-responsive *CsPti1-L* were also found in maize (Zou et al., 2006), soybean (Tian et al., 2004), and cucumber (Oh et al., 2014), which indicated the roles of phytohormone-responsive *cis*-elements.

### Post-translational Modification Mediated the Subcellular Localization of Ptis

The subcellular localization of Ptis is determined by their protein sequences and post-translational modifications (PTMs). The subcellular localizations of 11 Ptis from tomato, maize, rice, cucumber, and apple have been detected using the transient expression system in tobacco leaves, onion epidermal cells, tobacco suspension cells, and protoplasts of rice and maize (Table 1; Gu et al., 2002; Herrmann et al., 2006; Matsui et al., 2014; Oh et al., 2014; Schwizer et al., 2017; Wu et al., 2021).

**TABLE 1:** Experimentally verified Pti subcellular localization in different plant species.

Protein name	Protein type	Protein localization	Plant species	Method	Modification type/site or localization signal sequence	References
SlPti1a	Serine/threonine kinase	PM/periphery	Tobacco leaves	Transient expression	S-acylation of Cys^6^ and Cys^7^	Schwizer et al., 2017
		Cytoplasm	Onion epidermal cells	Transient expression	S-acylation of Cys^6^ and Cys^7^	Herrmann et al., 2006
SlPti1b	Serine/threonine kinase	PM/periphery	Tobacco leaves	Transient expression	S-acylation of Cys^6^ and Cys^7^	Schwizer et al., 2017
SlPti4	ERF transcription factor	Nucleus	Tobacco suspension cells	Transient expression	Nuclear localization sequences	Gu et al., 2002
SlPti5	ERF transcription factor	Nucleus	Tobacco suspension cells	Transient expression	Nuclear localization sequences	Gu et al., 2002
SlPti6	ERF transcription factor	Nucleus	Tobacco suspension cells	Transient expression	Nuclear localization sequences	Gu et al., 2002
ZmPti1a	Serine/threonine kinase	PM	Onion epidermal cells	Transient expression	Myristoylation and/or palmitoylation of Gly^2^, Cys^3^, Gly^2^/Cys^3^, and Cys^6^/Cys^7^	Herrmann et al., 2006
ZmPti1b	Serine/threonine kinase	Cytoplasm, nucleus	Onion epidermal cells	Transient expression	Acylation of Cys^3^, Cys^6^, or Cys^7^	Herrmann et al., 2006
ZmPti1c	Serine/threonine kinase	Cytoplasm	Onion epidermal cells	Transient expression	Acylation of Cys^6^ or Cys^7^	Herrmann et al., 2006
ZmPti1d	Serine/threonine kinase	Cytoplasm	Onion epidermal cells	Transient expression	Acylation of Cys^6^ or Cys^7^	Herrmann et al., 2006
OsPti1a	Serine/threonine kinase	PM	Rice protoplasts	Transient expression	Palmitoylation of Cys^6^ and/or Cys^7^	Matsui et al., 2014
CsPti1-L	Serine/threonine kinase	Cytoplasm	Tobacco leaves	Transient expression	-	Oh et al., 2014

*Sl, Solanum lycopersicum; Zm, Zea mays; Os, Oryza sativa; Cs, Cucumis sativus; Cys, cysteine; Gly, glycine; ERF, ethylene-response factors; PM, plasma membrane; / and -, unknown.*

In tomato, two homologous SlPti1s, namely, SlPti1a and SlPti1b, with 93% identity in protein sequence were localized to the plasma membrane (PM) and/or cell periphery, which were involved in ROS generation and resistance in response to *P. syringae* infection (Schwizer et al., 2017). However, when SlPti1a-GFP was transiently expressed in onion epidermal cells, GFP fluorescence was observed in both cytoplasm and nuclei (Herrmann et al., 2006). Interestingly, the cell periphery localization was presumed to determine by the S-acylation on cysteine (Cys)^6^ and Cys^7^ residue of Pti1a and Pti1b, because the tobacco transient expression of Cys^6^Ser/Cys^7^Ser substitutions of SlPti1a and SlPti1b exhibited their nuclear localizations (Schwizer et al., 2017). In addition, three tomato ERF transcription factors, namely, SlPti4, SlPti5, and SlPti6, were localized to the nucleus using the transient transformation assay in tobacco cells (Gu et al., 2002).

The protein PTMs are also implicated in the localization of four maize ZmPti1s (Herrmann et al., 2006). The ZmPti1a, ZmPti1b, ZmPti1c, and ZmPti1d were observed in PM, cytoplasm/nucleus, and cytoplasm using transient expression in onion epidermal cells (Herrmann et al., 2006). The predicted myristoylation and/or palmitoylation of Gly^2^, Cys^3^, Gly^2^/Cys^3^, and Cys^6^/Cys^7^ in ZmPti1a would affect its localization. The site-directed mutagenesis of the conserved Gly and Cys residues resulted in the migration of PM/cell periphery-localized kinase into the cytoplasm and/or nucleus (Herrmann et al., 2006). Besides, ZmPti1b, c, and d lack a Gly^2^ residue that is a potential target site of myristoylation and determine their PM localization (Herrmann et al., 2006). Additionally, both ZmPti1c and ZmPti1d contain a conserved N-terminal pair of arginine residues instead of a myristoylation signal, as well as conserved Cys^6^ or Cys^7^ with acylation, which led to their cytoplasm localization, but not nucleus localization (Herrmann et al., 2006). Similarly, the conserved Cys^6^ and Cys^7^ with predicted palmitoylation in rice OsPti1a determined its PM localization (Matsui et al., 2014). In addition, cytoplasm localization was observed in the cucumber Pti1-like protein (CsPti1-L) and apple MdPti1-L using transient expression in tobacco leaves and maize protoplasm, respectively (Oh et al., 2014; Wu et al., 2021).

Taken together, plant Pti1 proteins exhibited differential subcellular localization, owing to their varying competence for PM association being decided by the susceptibility to N-terminal myristoylation, palmitoylation, and/or S-acylation. Therefore, the PTM-mediated subcellular localization of Pti1s decides their diverse biological functions.

### Pti1a-Mediated Effector-Triggered Immunity and Patterns-Triggered Immunity Processes for Plant Disease Resistance

Higher plants have evolved the ability to recognize and resist pathogen infection (Andersen et al., 2018). Under the pathogen infection, plants activate PTI and ETI to initiate a series of defense responses, such as enhancement of ROS production, hypersensitive response (HR), induction of defense-related gene expression, and programmed cell death (PCD) at the infected site (Mehdy, 1994; Liu et al., 1999; Grant and Lamb, 2006; Sels et al., 2008). Microbial infection initially triggers the PTI after the pathogen-associated molecular patterns (PAMP) bind to the pattern recognition receptors (PRRs) at the cell surface, leading to ROS burst and the activation of signal transduction cascades, such as mitogen-associated protein kinases (MAPKs) and calcium-dependent protein kinases (CDPKs) signaling pathways (Nicaise et al., 2009; Tena et al., 2011). Once the pathogen breaks the PTI and secretes the effector proteins, the ETI is activated through the recognition between elicitors and their interacting cytoplasmic receptors (Dangl and Jones, 2001). In the ETI during the plant-pathogen interaction, the rapid activation of defense response is mediated by the specific recognition between the effector protein encoded by the avirulence (*avr*) gene of the pathogen and the resistance protein encoded by the corresponding resistance (*R*) gene of plants (Oh and Martin, 2011).

**FIGURE 3:**
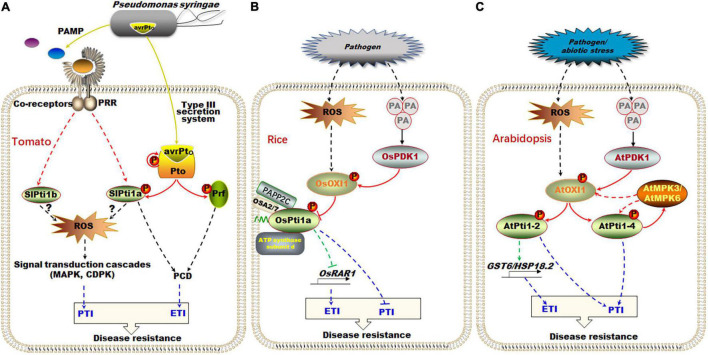
The regulation pathway of Pti1 in defense response and ROS signal in plant. (A) In tomato, the pathogen Pst injected the effector protein AvrPto into cell through type III secretion system. The Pto protein kinase can specifically recognize and interact with AvrPto, being activated through autophosphorylation, and then phosphorylated SlPti1a for triggering the programmed cell death in plant ETI response. Besides, SlPti1a and SlPti1b regulated ROS production and the downstream MAPK signaling pathway during the PTI process. (B) In rice, OsPti1a negatively regulated disease resistance by suppressing the expression of OsRAR1 in ETI during the infection of pathogenic bacteria. The pathogen infection also caused the independent ROS signal and PA signal and activated OsPDK1-OsOXI1-OsPti1a phosphorylation cascade in PTI. (C) In *Arabidopsis*, similar AtPDK1-AtOXI1-AtPti1-2/AtPti1-4 phosphorylation cascades also existed in response to PA signal. AtMPK3/AtMPK6 phosphorylated AtPti1-4 and OXI1, forming a feedback loop. AtPti1-2 promoted the expression of *GST6* and *HSP18.2* to scavenge ROS under oxidative stress. AvrPto, avirulence Pto; ETI, effector-triggered immunity; GST6, glutathione S-transferase 6; HSP, heat-shock proteins; MAPK, mitogen-associated protein kinases; OXI1, oxidative signal-inducible1; PA, phosphatidic acid; PDK1, 3-phosphoinositide-dependent protein kinase 1; Pst, *Pseudomonas syringae* pv. Tomato; PTI, pathogen-associated molecular patterns-triggered immunity; RAR1, required for Mla12 resistance. 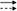: signal transformation; 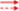: protein interaction; 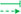: transcriptional regulation; 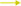: signal molecule from Pst DC3000; 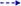: immune response; the broke line indicates indirect relationship, and the solid line indicates direct relationship.

It has been reported that tomato SlPti1a was interacted with Pto and was involved in the ETI process (Zhou et al., 1995). *Pto* gene encoding a serine/threonine protein kinase was originally obtained from wild tomato species (*Lycopersicon pimpinellifolium*) using map-based cloning and was identified as a disease resistance gene (Bogdanove and Martin, 2000). In tomato, Pto specifically recognized and interacted with the effector protein avirulence Pto (AvrPto) that was injected into the plant cell by the pathogen Pst through type III secretion system (Figure 3A). The interaction between Pto and AvrPto changed the conformation of Pto, and then, Pto switched to activating state from resting state through autophosphorylation or phosphorylation by other kinases (Dong et al., 2009). Subsequently, the Pto phosphorylated and activated its downstream effector SlPti1a, triggering the PCD in plant ETI defense response, which ultimately confers specific resistance to Pst (Figure 3A; Zhou et al., 1995; Sessa et al., 1998, 2000; Bogdanove and Martin, 2000). Besides, after being recognized and bound to AvrPto under Pst infection, Pto also can interact with Prf to synergistically activate the downstream ETI response (Figure 3A; Salmeron et al., 1996; Mucyn et al., 2006). Prf was another Pto interacting protein, belonging to the nucleotide-binding and leucine-rich repeat (NB-LRR) of the R protein family (Mucyn et al., 2006; Gutierrez et al., 2010). Interestingly, the Pto-Prf module-mediated ETI resistance was not obviously affected when the expressions of *SlPti1a* and *SlPti1b* were silenced simultaneously, which implies that the Pto-Prf module probably mediates a specific signaling pathway independent of the Pto-SlPti1a/SlPti1b module in ETI process (Figure 3A; Pedley and Martin, 2003; Schwizer et al., 2017).

Using a cell death suppression assay, it has been shown that SlPti1a and SlPti1b also play a regulatory role in ROS production and downstream MAPK signaling pathway during the early PTI process in tomato (Figure 3A; Schwizer et al., 2017). The tomato plants of simultaneously RNAi-silenced *Pti1a* and *Pti1b* were called hairpin-Pti1 (hpPti1) plants. Under flg22 and flgII-28 infection, the early ROS production in hpPti1 plants was reduced when compared with the wild-type tomato. The ROS was restored to the same level as it is in wild-type plants after *SlPti1a* and *SlPti1b* genes were transiently complemented to hpPti1 plants (Schwizer et al., 2017). However, how Pti1 regulates ROS production in the PTI process is still unclear (Figure 3A; Schwizer et al., 2017).

Additionally, the expression of both maize *ZmPti1* and soybean *GmPti1* was induced by SA, indicating that *ZmPti1* and *GmPti1* may function in the SA-dependent disease defense process (Tian et al., 2004; Zou et al., 2006). Besides, overexpressing cucumber *CsPti1-L* in tobacco enhanced resistance to necrotrophic pathogen *Botrytis cinerea*, which was attributed to the high expression of several defense-related genes, such as *NbPR1*, *NbPR2*, *NbPR5*, and *NbDEF2*. This implies that CsPti1-L would play a positive regulatory role in the pathogen defense response (Oh et al., 2014). The regulation mechanisms of Pti1 in maize, soybean, and cucumber to cope with diseases still need to be investigated.

### PDK1-OXI1-Pti1 Phosphorylation Cascade Mediates Disease and Oxidative Stress Responses

Rice *OsPti1a*, the homologous gene of tomato *SlPti1*, is a key negative regulator for ETI and PTI (Figure 3B; Takahashi et al., 2007; Matsui et al., 2014, 2015). Rice *ospti1a* mutant is a non-sense mutant plant, which was screened from a mutant line collection generated by rice endogenous retrotransposon Tos17 insertion (Hirochika et al., 2004). Under the infection of *Magnaporthe grisea*, the *ospti1a* mutants showed enhanced resistance to rice blast disease, while *OsPti1a* overexpressing plants increased the sensitivity to compatible pathogens (Takahashi et al., 2007).

For ETI, RAR1 (required for Mla12 resistance) was a required downstream factor for most known R proteins (e.g., NB-LRR proteins) (Figure 3B; Hammond-Kosack and Parker, 2003; Shen et al., 2003). The resistance of *ospti1a* mutants disappeared when the expression of *OsRAR1* was suppressed, which implied that the OsPti1a-mediated resistance to rice blast disease depended on *OsRAR1* in ETI defense response (Takahashi et al., 2007). Importantly, OsPti1a was localized at PM through its N-terminal myristoylation and/or palmitoylation modification (Figure 3B; Matsui et al., 2014). When the N-terminal domain was deleted, ΔN-OsPti1a mutants cannot complement the phenotype of *ospti1a* mutants, indicating that the negative regulatory function of OsPti1a on rice immune signals depends on the N-terminal palmitoylation-mediated PM localization (Matsui et al., 2014). Moreover, immunoprecipitation combined with mass spectrometry revealed that OsPti1a probably can interact with a variety of disease-related proteins on the PM, such as PM H^+^-ATPase isoform2 (OSA2), OSA7, vacuolar ATP synthase subunit d, and phytochrome-associated phosphatase type 2C (PAPP2C) (Figure 3B; Matsui et al., 2015). However, the interaction and regulation mechanism of these candidate proteins interacting with OsPti1a still needs to be verified.

The infection of pathogenic bacteria usually triggered two relatively independent signals, namely, ROS signal and phosphatidic acid (PA) signal, in plant cells (Figure 3B; Testerink and Munnik, 2005; Yamaguchi et al., 2005). In the ROS-mediated defense response, oxidative signal-inducible1 (OXI1) is a positive regulator, which belongs to the AGC VIII family encoding a serine/threonine protein kinase (Rentel et al., 2004). It was reported that OXI1 was activated by ROS signal, but the mechanism in rice is still unknown (Rentel et al., 2004). In the PA signaling pathway, the 3-phosphoinositide-dependent protein kinase 1 (PDK1), another member of the AGC families, is specifically activated by the phospholipase D (PLD)-generated PA signals (Matsui et al., 2015). The activated OsPDK1 was interacted with phosphorylated OsOXI1 (Matsui et al., 2010a), and then, OsOXI1 phosphorylated OsPti1a at its conserved Thr-233 residue, thereby inhibiting the negative regulatory function of OsPti1a during defense responses (Figure 3B; Matsui et al., 2010b). This implies that the OsPDK1-OsOXI1-OsPti1a phosphorylation cascade plays an important role in activating PTI in rice (Matsui et al., 2015).

Similarly, the interaction of AtPti1-2/AtPti1-4 and AtOXI1 also integrated the independent ROS and PA signal in *Arabidopsis* (Figure 3C; Anthony et al., 2006; Forzani et al., 2011). AtOXI1 has been reported to be activated by ROS during plant immune defense (Anthony et al., 2004; Rentel et al., 2004). AtOXI1 has been screened to interact with AtPti1-1, AtPti1-2, AtPti1-3, and AtPti1-4 through yeast two-hybrid system (Anthony et al., 2006; Petersen et al., 2009; Forzani et al., 2011). Among them, AtPti1-2 was proved to be phosphorylated and activated by AtOXI1, subsequently promoting the expression of ROS scavenging-related genes (e.g., *GST6* and *HSP18.2*) in response to oxidative stress (Figure 3C; Anthony et al., 2006). Moreover, AtOXI1 was also interacted with and phosphorylated by the upstream AtPDK1 in response to the PA signal generated by environmental stress, including osmotic and temperature stress, oxidative stress, and pathogen stress (Figure 3C; Anthony et al., 2004, 2006).

In addition, AtPti1-4 was also phosphorylated and activated by AtOXI1 in response to oxidative stress (Forzani et al., 2011). Interestingly, AtPti1-4 was directly interacted with AtMPK3/AtMPK6 and phosphorylated by them (Forzani et al., 2011). It has been reported that the AtOXI1 was positioned upstream of AtMPK3 and AtMPK6, and the full activation of AtMPK3 and AtMPK6 required AtOXI1 activity in *Arabidopsis* under abiotic stresses (Figure 3C; Rentel et al., 2004). However, AtOXI1 was detected to be phosphorylated by AtMPK3 and AtMPK6 *in vitro*, although no direct interaction was detected *in vivo* (Forzani et al., 2011). Thus, it should be speculated that AtMPK3 and AtMPK6 probably act as a downstream factor for feedback regulation of AtOXI1 and subsequently AtPti1-4 in response to oxidative stress (Figure 3C; Yoshioka et al., 2003; Forzani et al., 2011).

### OXI1-PTI1 Module Regulates Tip Growth of Pollen Tube and Root Hair

The MRI is a member of the *Arabidopsis* Pti1 protein kinase family, which is preferentially and highly expressed in pollen grains, pollen tubes, and roots of *Arabidopsis* (Boisson-Dernier et al., 2015). The MRI mutant allele *mri-4* was generated through transposable elements of the Ac/Ds system (Sundaresan et al., 1995). The *Arabidopsis mri-4* plants exhibited pollen tube burst and abnormal growth upon pollen germination, suggesting that MRI plays an important role in pollen tube growth. Besides, the root hair elongation was also severely affected in *Arabidopsis mri-4* mutant, which was similar to the phenotype of the delayed root hair elongation in *oxi1* mutants (Rentel et al., 2004; Liao et al., 2016). Moreover, MRI was proved to interact with root hair development-related OXI1 *in vivo* and *in vitro* using Y2H, luciferase complementation (LUC), and co-immunoprecipitation (co-IP) detection and was phosphorylated by OXI1 (Liao et al., 2016). Therefore, the OXI1-MRI module may participate in the tip growth of the pollen tube and the root hair. In apple (*M. domestica*), the MdOXI1-MdPTI1L module was supposed as a “stop” signal upon the pollen tube growth in S-RNase-mediated gametophytic self-incompatibility (Wu et al., 2021). In this process, self-S-RNase induced the ROS increase in pollen tubes, triggering ROS-responsive MdOXI1 bound to and phosphorylated MdPTI1L, and the phosphorylated MdPTI1L subsequently inhibited the pollen tube growth (Wu et al., 2021). The downstream genes/proteins and the regulation mechanism of OXI1-PTI1 module in pollen tubes and root hairs need to be further investigated.

### Pti1 Is Involved in the Diverse Abiotic Stresses

The homologs of Pti in maize, soybean, and cucumber were involved in salts, drought, and low temperature, respectively (Staswick, 2000; Tian et al., 2004; Anthony et al., 2006; Herrmann et al., 2006; Oh et al., 2014). The expression of *ZmPti1* gene in maize was induced under high salts and low temperature, but not induced by ABA, which indicated that ZmPti1 was involved in salt and low-temperature stresses through an ABA-independent pathway (Zou et al., 2006). The expression of cucumber *CsPti1-L* was induced by salt stress (Zou et al., 2006; Oh et al., 2014). The photosynthetic capacity and salt tolerance of *CsPti1-L*-overexpressing tobacco were increased, which was correlated with the high expression of the stress-related gene *dehydrin* in the transgenic tobacco (Oh et al., 2014).

### Pti4, Pti5, and Pti6 Function as Transcription Factors for Regulating Effector-Triggered Immunity Process

**FIGURE 4:**
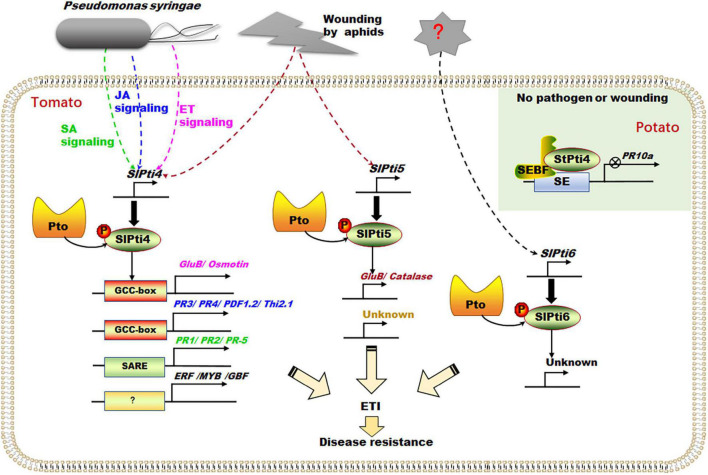
Transcription factors Pti4, Pti5, and Pti6 regulate the ETI process. Pst infection can induce the expression of transcription factor *SlPti4* through different phytohormones (e.g., ethylene, SA, and JA) signal pathways, which regulate the expression of a series of PR genes containing GCC-box *cis*-acting element, SARE *cis*-acting element, and other transcription factors (e.g., ERF, MYB, and GBF) containing other *cis*-acting elements. Pst infection and wounding of tomato aphids can induce the expression of transcription factor *SlPti5*, which subsequently accelerates the expression of several defense-related genes (e.g., *GluB* and *Catalase*). In potato, under no wound or effector conditions, StPti4 interacted with SEBF to form an inhibitory complex to inhibit the expression of *PR-10a*. ETI, effector-triggered immunity; ERF, ethylene-response factors; GBF, G-box factor; *GluB*, glucanase B; JA, jasmonate; PR, pathogenesis-related; Pst, *Pseudomonas syringae* pv. Tomato; SA, salicylic acid; SARE, salicylic acid-responsive element; SEBF, silencer element-binding factor. The broke line indicates indirect relationship, and the solid line indicates direct relationship.

SlPti4, SlPti5, and SlPti6 belong to the ERF transcription factor family. They contain a DNA-binding domain, an acidic residue region for transcription activation, and a nuclear localization signal sequence, which can interact with tomato Pto protein kinase (Zhou et al., 1997). SlPti4, SlPti5, and SlPti6 regulate the expression of a series of pathogenesis-related (PR) genes by specifically binding to their GCC-box *cis*-acting elements and exhibit diverse regulation patterns (Figure 4; Zhou et al., 1997; Gu et al., 2000, 2002; Wu et al., 2002).

Pathogen-induced phytohormones (e.g., ethylene, SA, and JA) regulated *SlPti4* expression, triggering the downstream signal pathways (Figure 4; Thomma et al., 1998; Gu et al., 2000, 2002). *SlPti4* was rapidly ethylene-induced and was necessary for the activation of ethylene-regulated *PR* genes containing GCC-box, such as *glucanase B* (*GluB*) and *osmotin* (*Osm*) (Figure 4; Gu et al., 2000). Importantly, the phosphorylation of SlPti4 in nuclei by Pto would enhance the specific binding of SlPti4 to the GCC-box *cis*-acting element in promoters of *PR* genes and further promote *PR* expression, positively regulating the defense response (Figure 4; Gu et al., 2000). Besides, *SlPti4* can be upregulated by SA under Pst infection and moderate damage of wounding (Figure 4; Gu et al., 2002). The heterologous expression of *SlPti4* in *Arabidopsis* activated the expression of various SA-regulated genes (e.g., *PR1* and *PR2*) containing SA-responsive element (SARE) and JA/ethylene-regulated *PR* genes (e.g., *PR3*, *PR4*, *PDF1.2*, and *Thi2.1*) containing GCC-box (Kumar, 2014). Thus, SlPti4 was involved in SA- and JA/ethylene-dependent signaling pathways to enhance the pathogen defense (Figure 4; Thomma et al., 1998; Gu et al., 2002). Moreover, SlPti4 can also bind to the promoters of other transcription factors (e.g., *ERF*, *MYB*, and *GBF*) that do not contain GCC-box, indicating SlPti4 indirectly regulates the downstream gene expression together with other transcription factors (Figure 4; Chakravarthy et al., 2003). Interestingly, under no wounding or effector-induced conditions, potato (*Solanum tuberosum*) StPti4 was recruited by and interacted with the repressor silencer element-binding factor (SEBF) to form an inhibitory complex, which binds to the promoter of *PR-10a* to inhibit its expression (Figure 4). This suggests that StPti4, and its homologous SlPti4, would function as a transcription repressor under normal conditions (González-Lamothe et al., 2008).

*SlPti5* was involved in the resistance to wounding of tomato aphids and Pst infection (Figure 4; Gu et al., 2000; He et al., 2001; Wu et al., 2015). *SlPti5* was induced slightly in tomato by wounding, but not changed under ethylene treatment (Gu et al., 2000). Besides, SlPti5 played an important role in aphid resistance (Wu et al., 2015). *SlPti5*-silenced tomato plants impaired the resistance to tomato aphid population (Wu et al., 2015), while potato aphid infection can upregulate the expression of *SlPti5* independent of ethylene signaling (Wu et al., 2015). In addition, overexpressing *Pti5* tomato plants enhanced the resistance to Pst infection by accelerating the expressions of several defense-related genes (e.g., *GluB* and *Catalase*) (Figure 4; He et al., 2001). Moreover, SlPti4/SlPti5/SlPti6-overexpressed tomato plants showed enhanced resistance to Pst (Wang et al., 2021). However, Gu et al. (2000) reported that SlPti6 was not responsive to ethylene, SA, Pst infection, and insect wounding. The fine-tuned phytohormone-mediated molecular mechanisms of SlPti4, SlPti5, and SlPti6 modulations are still unclear. The downstream target genes of SlPti5 and SlPti6 and their working models need to be investigated by biochemistry and molecular genetic strategy.

### SlPti4 Is Involved in the Regulation of Abscisic Acid-Mediated Development and Stress Tolerance

Tomato SlPti4 participates in fruit ripening and seed germination by regulating ABA metabolism. *SlPti4* RNAi transgenic fruits showed increased ABA level caused by the upregulation of *SlNCED1* in ABA biosynthesis at mature green (MG) and breaker (B) stages and the downregulation of *SlCYP707A2* in ABA degradation throughout fruits ripening process. Meanwhile, the expression patterns of the ABA receptor family *SlPYL* genes were varied. Besides, the expression of *PYL1*, *PYL2*, *PYL5*, *PYL6*, and *PYL9* was upregulated during this process except in the B stage, while the expression of *PYL4*, *PYL7*, and *PYL10* was downregulated in *SlPti4*-RNAi fruits during ripening. However, the molecular mechanisms of SlPti4-mediated ABA signaling and metabolism upon fruit ripening are still unclear (Sun et al., 2018).

*SlPti4*-RNAi seeds germinated earlier than seeds of wild type (WT) with ABA treatments. The ABA content and expression of *SlNCED1/2* in dry seeds were markedly lower than that in the WT seeds. Moreover, the expression of several *SlPYLs* (i.e., *SlPYL3*, *SlPYL4*, *SlPYL6*, *SlPYL9*, and *SlPYL10*) in *SlPti4*-RNAi seeds was significantly lower than that in the WT seeds. Additionally, the expressions of two representative ABA response genes, namely, *SlABI3* and *SlABI5*, were downregulated in transgenic seeds compared with those in WT seeds under ABA treatments. These indicate that the downregulation of *SlPti4* reduced the ABA sensitivity in seeds (Sun et al., 2018).

In addition, the tomato transcription factor SlPti4 plays a role in response to drought stress. Silencing SlPti4 resulted in reduced drought resistance and less accumulation of ABA content, but high ethylene release. Meanwhile, the expression levels of most ABA receptor *PYL* genes (i.e., *SlPYL5*, *SlPYL6*, *SlPYL7*, *SlPYL8*, *SlPYL9*, *SlPYL10*, and *SlPYL11*) were lower than that in the wild type, which indicated that SlPti4 may enhance the drought resistance by regulating ABA content and perception (Sun et al., 2018).

Recently, SlPti4/5/6-overexpressed tomato plants showed accelerated ripening of fruits but no change in the flowering time, the seed-setting rate, and seeds development (Wang et al., 2021). Moreover, the expression of ethylene synthesis genes *ACS2* and *ACS4* (1-aminocyclopropane-1-carboxylate synthase) and ripening-related transcription regulator *CNR* (colorless non-ripening) increased in the mature green stage of the fruits of SlPti4/5/6-overexpression lines (Wang et al., 2021). These results suggested that SlPti4/5/6 functions as a transcription activator to regulate ripening-related genes through the ethylene signaling pathway.

## Conclusion and Perspectives

Ptis mainly refer to a group of protein kinases, which can be interacted with and phosphorylated by Pto. Various PM-localized Pti1s (e.g., SlPti1a, OsPti1a, AtPti1-4, and AtPti1-2) were phosphorylated by Pto for PTI and ETI in tomato under Pst infection or by OXI1 for the modulation of downstream gene expression in rice and *Arabidopsis* to cope with disease and stress. Additionally, some transcription factors were previously defined as Pti4, Pti5, and Pti6, which were involved in the regulation of JA, SA, and ethylene signaling pathways. These transcription factors were phosphorylated by Pto and then bind to the GCC-box, SARE of the downstream genes for initiating their expressions involved in Pst infection, insect wounding, and other stress responses, respectively. All these indicate that Ptis are critical regulators in plant development, disease resistance, and stress response. However, how they function as kinase to perceive upstream hormone and ROS signals and to modulate downstream kinase signaling pathways for enhancement of disease insistence is still unclear. Therefore, biochemistry, molecular genetics, and multi-omics studies are necessary for further discovering the diverse and fine-tuned functions of Ptis in plant development and stress tolerance.

## Author Contributions

MS and LQ wrote the manuscript with suggestions by ZQ and SD. YL, HZ, YZ, YQ, YM, MZ, and XD performed the bioinformatics analysis. All authors contributed to the article and approved the submitted version.

## Conflict of Interest

The authors declare that the research was conducted in the absence of any commercial or financial relationships that could be construed as a potential conflict of interest.

## Publisher’s Note

All claims expressed in this article are solely those of the authors and do not necessarily represent those of their affiliated organizations, or those of the publisher, the editors and the reviewers. Any product that may be evaluated in this article, or claim that may be made by its manufacturer, is not guaranteed or endorsed by the publisher.

## Footnotes

1
https://www.ncbi.nlm.nih.gov

2
http://bioinformatics.psb.ugent.be/webtools/plantcare/html/
